# A Novel Bicistronic Adenovirus Vaccine Elicits Superior and Comprehensive Protection Against BVDV

**DOI:** 10.3390/microorganisms14020378

**Published:** 2026-02-05

**Authors:** Mingguo Xu, Chuangfu Chen, Hengyun Gao, Hao Guo, Xueyu Tao, Huan Zhang, Yong Wang, Zhongchen Ma, Zhen Wang, Ningning Yang, Hui Zhang

**Affiliations:** 1 College of Animal Science and Technology, Shihezi University, Shihezi 832000, China; xumingguo@xjshzu.com (M.X.); chuangfu_chen@163.com (C.C.); 15936591679@163.com (H.G.); 15083016851@163.com (H.G.); taoxueyu@xjshzu.com (X.T.); likezhanghuan@aliyun.com (H.Z.); yongwang@shzu.edu.cn (Y.W.); zhongchen_ma@163.com (Z.M.); wzhen2018@shzu.edu.cn (Z.W.); 2 College of Animal Science and Technology, Xinyang Agriculture and Forestry University, Xinyang 464000, China

**Keywords:** bovine viral diarrhea virus, bicistronic recombinant adenovirus, neutralizing antibody, viral load, protective efficacy

## Abstract

Bovine viral diarrhea virus (BVDV) is a major pathogen inflicting substantial economic losses on the global cattle industry. To develop a more effective vaccine, we constructed two novel bicistronic recombinant adenoviruses, rAdV-I E0+I E2 and rAdV-I E2+II E2, and systematically evaluated their immunogenicity and protective efficacy in BALB/c mice. Both vaccine candidates, particularly rAdV-I E2+II E2, provoked a robust and rapid neutralizing antibody response that was significantly superior to a commercial inactivated vaccine. They also elicited a potent Th1-skewed cellular immune response, as indicated by significantly higher IFN-γ secretion, and a balanced profile of BVDV-specific IgG and its subclasses. Upon BVDV challenge, immunization with both recombinant vaccines, especially rAdV-I E2+II E2, resulted in a comprehensive reduction in viral loads across all tested tissues (blood, spleen, lungs, kidneys, and small intestine), demonstrating broader protection than the inactivated vaccine. Concordantly, histopathological analysis confirmed that vaccination preserved the normal architecture of the duodenum and spleen, preventing the significant pathological damage observed in the rAdV-empty negative control group. Our findings demonstrate that these adenovirus-vectored vaccines, particularly rAdV-I E2+II E2, induce a multifaceted and protective immune response, highlighting their promise as superior candidates against BVDV.

## 1. Introduction

Bovine viral diarrhea virus (BVDV), a member of the genus *Pestivirus*, is a major pathogen responsible for substantial economic losses in the global cattle industry [1,2]. Infection can lead to a wide spectrum of clinical outcomes, including reproductive failure, respiratory and digestive diseases, and the birth of persistently infected (PI) animals that serve as perpetual reservoirs for viral transmission [3,4]. The control and eradication of BVDV heavily rely on effective vaccination. Conventional inactivated and modified-live virus (MLV) vaccines are widely used but have limitations [5,6]. Inactivated vaccines often provide short-lived immunity, while MLV vaccines carry potential risks such as reversion to virulence and complicate disease surveillance due to the inability to differentiate infected from vaccinated animals (DIVA).

Novel vaccine platforms, particularly viral-vectored vaccines, have emerged as promising alternatives. Recombinant adenovirus (rAdV) vectors are attractive due to their safety, high transduction efficiency, and capacity to induce robust humoral and cellular immune responses [7,8,9]. Their ability to accommodate large or multiple foreign antigens makes them ideal for developing multivalent vaccines [10,11,12]. Furthermore, adenoviral vectors have demonstrated strong immunogenicity in numerous species, making them a versatile platform for veterinary vaccine development [13,14].

The immunogenicity of BVDV vaccines is critically dependent on the key structural proteins included in the formulation. The E2 glycoprotein is the major immunodominant antigen, capable of eliciting potent virus-neutralizing antibodies, which are considered a primary correlate of protection [15,16,17]. Additionally, the E0 (Erns) glycoprotein, possessing intrinsic RNase activity, is also a target for neutralizing antibodies and may play a role in modulating the host immune response [18,19,20]. However, most prior studies using adenoviral vectors or subunit platforms have focused on expressing single BVDV antigens [15,16,17,21,22]. The potential synergistic effects of combining these key antigens from different BVDV genotypes within a single bicistronic adenovirus vector to elicit broader and more potent immunity remain an area of active investigation.

In this study, we hypothesized that a novel bicistronic adenovirus vector co-expressing key antigenic proteins from BVDV-1 and BVDV-2 would elicit a superior protective immune response compared to conventional inactivated vaccines and previously reported single-antigen platforms. To test this, we constructed and characterized two novel bicistronic recombinant adenoviruses: rAdV-I E0+I E2 (expressing BVDV-1 E0 and E2) and rAdV-I E2+II E2 (expressing BVDV-1 E2 and BVDV-2 E2). We systematically evaluated their immunogenicity, including humoral and cellular immune responses, and their protective efficacy against a BVDV-1 challenge in a BALB/c mouse model, benchmarking their performance against a commercially available inactivated vaccine.

## 2. Materials and Methods

### 2.1. Experimental Mice

Fifty female BALB/c mice (6–8 weeks old, mean weight 20 ± 5 g) were purchased from the Animal Experiment Center of Xinjiang Medical University (Urumqi, China). Mice were housed under a 12 h light-dark cycle at 20 °C and 55% relative humidity with ad libitum access to food and water. All animal experiments were approved by the Biology Ethics Committee of Shihezi University (Approval No. A2023-217).

### 2.2. Cell Lines and Virus Culture

Human embryonic kidney (293A) cells and Madin–Darby bovine kidney (MDBK) cells were obtained from the National Collection of Authenticated Cell Lines (Shanghai, China). Cells were maintained in Dulbecco’s modified Eagle’s medium (DMEM; Gibco, New York, NY, USA) supplemented with 10% fetal bovine serum (FBS; Gibco, USA) at 37 °C in a 5% CO_2_ atmosphere. BVDV-1 strains, identified and maintained by the Zoonotic Diseases Laboratory at Shihezi University, were propagated in MDBK cells. The 50% tissue culture infective dose (TCID_50_) was determined via the Reed–Muench method [23].

### 2.3. Construction and Characterization of rAdV-I E0+I E2 and rAdV-I E2+II E2

The nucleotide sequences encoding BVDV-1 E0 (amino acids 271–497; GenBank accession no. QCQ84262.1), BVDV-1 E2 (aa 693–1066; QCQ84262.1), and BVDV-2 E2 (aa 693–1064; ACQ83621.1) were optimized based on codon usage preferences for eukaryotic expression systems. The optimized sequences, flanked by SalI/EcoRV or SalI/XhoI restriction sites, were synthesized by General Biosystems and subsequently cloned into the pAdTrack-CMV shuttle vector (MiaoLing Plasmid, Wuhan, China). This generated the recombinant shuttle plasmids, designated pAdTrack-I E0+I E2 and pAdTrack-I E2+II E2. These recombinant plasmids were linearized with PmeI and then transformed into *E. coli* BJ5183 (MiaoLing Plasmid, China) competent cells containing the adenoviral backbone plasmid pAdeasy-1 (MiaoLing Plasmid, China) for homologous recombination in vivo. The successfully recombined adenoviral plasmids were named PAdEasy-I E0+I E2 and PAdEasy- I E2+II E2. Following linearization with PacI, these plasmids were transfected into 293A cells for virus packaging, yielding the recombinant adenoviruses rAdV-I E0+I E2 and rAdV-I E2+II E2. A control adenovirus lacking the target gene sequences, rAdV-empty, was generated using the same protocol.

To assess viral genetic stability, recombinant adenoviruses from passages 5, 10, and 15 were harvested. Viral samples were treated with 10 μL of proteinase K, incubated at 55 °C for 1 h, and then heat-inactivated at 100 °C for 5 min. The treated samples served as templates for PCR amplification of the target gene sequences using specific primers (Table 1). The PCR products were analyzed by 1% agarose gel electrophoresis. The DNA bands corresponding to the target genes were excised and sent to Beijing RuiBoXingKe Biotechnology Co., Ltd. (Beijing, China) for sequencing verification.

To analyze the expression of the I E0+I E2 and I E2+II E2 proteins, supernatants from transfected cells were harvested, separated by 12% SDS-PAGE, and transferred onto 0.45 μm polyvinylidene fluoride (PVDF) membranes (Millipore, Burlington, MA, USA). The membranes were blocked at room temperature for 2 h with TBST containing 5% skimmed milk. Subsequently, they were incubated overnight at 4 °C with BVDV-positive serum (dilution, 1:500). After washing with TBST, the membranes were incubated for 2 h with HRP-conjugated rabbit anti-bovine IgG secondary antibody (dilution, 1:4000; Solarbio, Beijing, China). Finally, protein bands were visualized using an ECL substrate, and images were captured using a ProteinSimple gel imaging system.

### 2.4. Vaccination Protocol and Sample Collection

**Figure 1:**
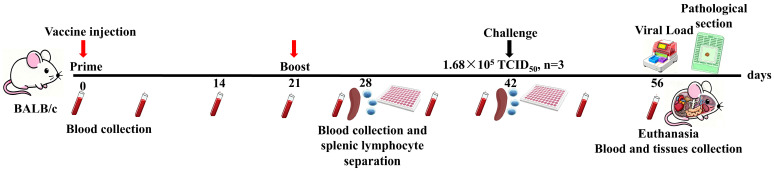
Schematic timeline of the immunization and challenge protocol. The diagram outlines: prime-boost immunization on days 0 and 21; immune monitoring (splenocyte and serum sampling); BVDV-1 challenge at day 42; and terminal tissue collection at day 14 post-challenge for viral load measurement and histopathological examination.

Fifty female BALB/c mice (6–8 weeks old) were randomly allocated into five groups (*n* = 10) to form the following cohorts: two experimental groups, two control groups [PBS (blank control) and rAdV-empty (negative control, NC)], and a commercial inactivated vaccine (TECON, Nanchang, China) as the positive control (PC). All mice were immunized subcutaneously following a protocol adapted from Elahi et al. [24] (Table 2). Following each immunization, the mice were monitored daily for 48 h for adverse reactions, with the protocol adjusted as necessary. Blood samples were collected at the time points indicated in Figure 1. Serum was separated by centrifugation and stored at −20 °C for subsequent antibody analysis.

### 2.5. Enzyme-Linked Immunospot (ELISpot) Assay

Cellular immunity was assessed using the IFN-γ ELISpot assay on days 28 and 42 post-primary immunization [25,26]. Briefly, splenocytes were isolated from three randomly selected mice per group. Cells (1 × 10^6^/well) were seeded in pre-coated plates and stimulated with the corresponding antigen (10 μg/mL; experimental groups), Concanavalin A (ConA; 10 μg/mL; positive control), rAdV-empty (negative control) or PBS (blank control). After 30 h of incubation, spot-forming cells (SFCs) were visualized according to the manufacturer’s instructions (Mabtech, Nacka Strand, Sweden).

### 2.6. Enzyme-Linked Immunosorbent Assay (ELISA)

Specific IgG, IgG1, and IgG2a antibodies in serum samples were quantified using an indirect ELISA as previously described [26,27]. Briefly, either I E0+I E2 or I E2+ II E2 protein antigens were diluted according to established protocols and immobilized onto 96-well ELISA plates (100 µL per well), followed by overnight incubation at 4 °C. After discarding the coating solution, the wells were washed twice with PBST (phosphate-buffered saline with 0.05% Tween 20) and dried. The wells were blocked with 200 µL of 5% nonfat dry milk (BD, Franklin Lakes, NJ, USA) and incubated at 37 °C for 2 h. After two additional washes with PBST, diluted serum samples (100 µL) were added and incubated at 37 °C for 1 h. After five washes with PBST, 100 µL of HRP-conjugated goat anti-mouse IgG, IgG1, or IgG2a (Proteintech, Wuhan, China) was added and incubated at 37 °C for 1 h. Following five washes with PBST, 100 µL of TMB substrate solution (Solarbio, China) was added and incubated for 15 min in the dark. The reaction was terminated by adding 50 µL of stop solution (Solarbio, China), and the optical density (OD) at 450 nm was measured using a microplate reader. To ensure specificity, control wells were prepared without serum samples, and the absorbance in these wells was subtracted from the experimental values.

### 2.7. Virus Neutralization Test (VNT)

The VNT is considered the gold standard for both serodiagnosis and vaccine efficacy evaluation of BVDV and Classical Swine Fever Virus (CSFV) [28,29]. In this study, the BVDV-1 strain was selected as the challenge virus due to its widespread geographic and temporal distribution [30,31]. Serum samples were heat-inactivated at 56 °C for 30 min. Two-fold serial dilutions of the serum (1:2 to 1:256) were prepared in DMEM, and an equal volume of the BVDV-1 virus (100 TCID_50_) was added to each serum dilution. The virus-serum mixture was incubated at 37 °C for 1 h. Subsequently, the mixtures were transferred onto MDBK cell monolayers and adsorbed at 37 °C for 2 h. After adsorption, the inoculum was removed, and the cells were cultured in growth medium with 1% FBS at 37 °C in a 5% CO_2_ atmosphere for 5–7 days. Cytopathic effects (CPE) were observed daily and scored using an inverted microscope (Nikon, Tokyo, Japan). The neutralizing antibody titer was determined as the dilution that inhibited 50% of CPE and calculated using the Reed–Muench method [23].

### 2.8. Viral Challenge Experiment

Based on epidemiological data indicating that BVDV-1 strains exhibit the highest incidence in both swine and cattle populations [30,32], the BVDV-1 strain, a representative subtype, was selected for the murine challenge study. The challenge dose and route of administration were determined from a prior efficacy study [26]. Accordingly, on day 42 post-primary immunization, mice were challenged via intraperitoneal injection with 1.68 × 10^5^ TCID_50_ of the virus.

### 2.9. Sample Collection and Viral Load Quantification

On day 14 post-challenge, blood was collected from mice via retro-orbital bleeding using glass capillaries and transferred into anticoagulant-treated tubes. Immediately following blood collection, the mice were euthanized by cervical dislocation. Major organs, including the heart, liver, spleen, lungs, kidneys, and small intestine, were aseptically harvested. Total RNA was extracted from each tissue sample using a commercial kit according to the manufacturer’s instructions and reverse-transcribed into complementary DNA (cDNA). The viral load in each tissue was quantified by reverse transcription quantitative PCR (RT-qPCR). The absolute viral load was determined by interpolating cycle threshold (Ct) values against a standard curve generated from serially diluted plasmids of known concentration, as previously described [26].

### 2.10. Histopathological Analysis

Duodenum and spleen tissues were fixed in 4% paraformaldehyde for 24–48 h, processed through standard dehydration (graded ethanol) and clearing (xylene) steps, and embedded in paraffin. Serial sections were cut at 4 μm thickness and stained with hematoxylin and eosin (H&E). The stained sections were examined under a light microscope (Nikon, Japan) and imaged using a digital camera.

### 2.11. Statistical Analysis

Statistical analyses were performed with GraphPad Prism 8.0 (GraphPad Software, Inc., San Diego, CA, USA) using one- or two-way ANOVA, as appropriate. Following a significant ANOVA result (*p* < 0.05), post hoc comparisons between relevant groups were performed to identify specific differences. A *p*-value < 0.05 was considered statistically significant. The sample size (number of biological replicates, n) for each experiment is explicitly stated in the corresponding methods sections above. Viral loads in tissue samples and TCID_50_ values were calculated separately using Microsoft Excel (Microsoft Corporation, Redmond, WA, USA). All in vitro experiments were performed in triplicate, and data are presented as means ± standard deviations (SDs).

### 2.12. Illustration Generation

Schematic components in Figure 1 (including depictions of a mouse, PCR instrument, blood collection tube, 96-well plate, and tissue block) were created using the artificial intelligence (AI) image generation tool “Doubao” (Version 2025; ByteDance Ltd., Beijing, China) based on detailed textual descriptions provided by the authors. All AI-generated outputs were critically evaluated, meticulously edited, and integrated by the authors, who assume full responsibility for the accuracy and presentation of the final figures.

## 3. Results

### 3.1. Construction, Characterization, and Identification of rAdV-I E0+I E2 and rAdV-I E2+II E2

**Figure 2:**
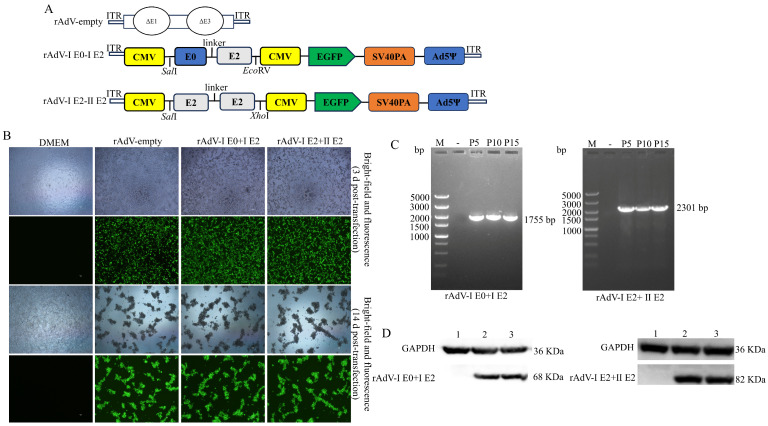
Construction and in vitro characterization of recombinant adenoviruses rAdV-I E0+I E2 and rAdV-I E2+II E2. (**A**) Schematic diagrams of the recombinant adenoviral genome structures of rAdV-empty, rAdV-I E0+I E2 and rAdV-I E2+II E2. (**B**) EGFP fluorescence and bright-field images of 293A cells at 3 and 14 d post-transfection. DMEM-treated cells served as mock controls. (**C**) PCR verification of recombinant viral genomes across serial passages. “P” indicates viral passage; “-” indicates negative control. (**D**) Western blot analysis confirming expression of the expected fusion proteins, I E0+I E2 and I E2+II E2. GAPDH was used as the loading control.

The construction strategy for the recombinant bicistronic adenoviruses is shown in Figure 2A. The fusion genes encoding BVDV-1 E0-E2 (rAdV-I E0+I E2) and BVDV-1 E2-BVDV-2 E2 (rAdV-I E2+II E2) were cloned into the pAdTrack-CMV shuttle vector and recombined with the adenoviral backbone in vitro (Figure S1A). The recombinant plasmids were verified by *Pac*I restriction analysis (Figure S1B). Following PacI digestion, the constructs were transfected into 293A cells. By day 3, robust EGFP expression was observed in cells transfected with rAdV-empty, rAdV-I E0+I E2 and rAdV-I E2+II E2, but not in DMEM-treated controls (Figure 2B). By day 14, extensive cytopathic effects (CPE), including cell rounding, shrinkage, and detachment, were evident in transfected cultures (Figure 2B). PCR analysis of serially passaged viral supernatants confirmed the stable maintenance of the target genes (Figure 2C). Sequencing verified the integrity of the cloned sequences, demonstrating genetic stability. Western blot analysis detected specific bands at approximately 68 kDa and 82 kDa, corresponding to the expected sizes of the I E0+I E2 and I E2+II E2 fusion proteins, respectively (Figure 2D).

### 3.2. Recombinant Adenovirus Vaccines Elicit Robust and Durable Antigen-Specific IFN-γ Responses

**Figure 3:**
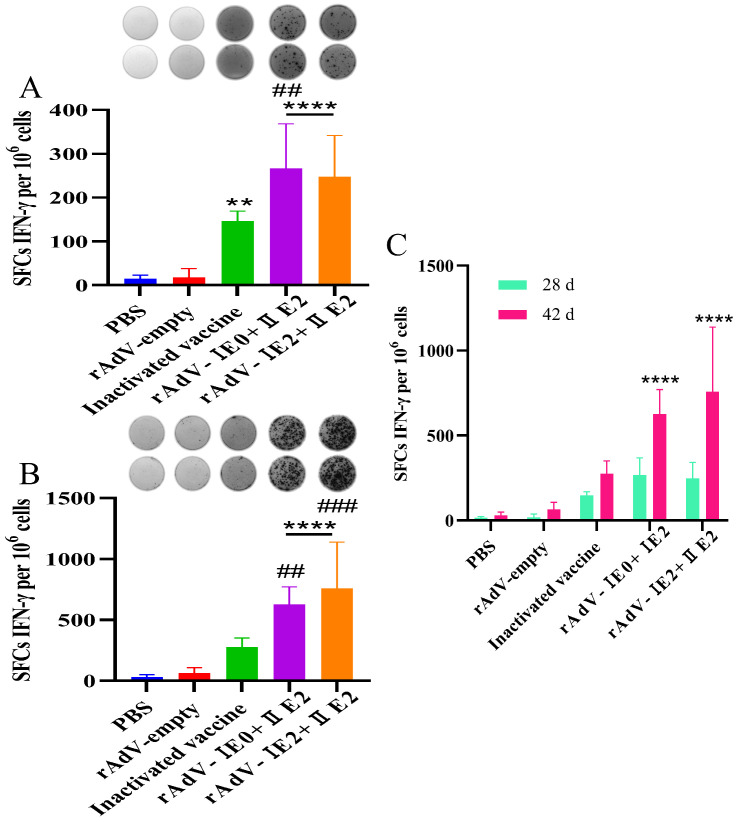
Recombinant adenovirus vaccines induce potent antigen-specific IFN-γ responses. BALB/c mice were immunized with either rAdV-I E0+I E2 or rAdV-I E2+II E2 vaccine. A commercial inactivated vaccine and rAdV-empty served as PC and NC, respectively. Splenocytes were isolated at days 28 and 42 post-immunization and re-stimulated with the corresponding antigen. IFN-γ-secreting cells were quantified by ELISpot (SFCs per 106 cells). (**A**,**B**) Representative ELISpot wells (**top**) and quantitative analysis (**bottom**) of IFN-γ^+^ SFCs at day 28 (**A**) and day 42 (**B**). (**C**) Comparison of IFN-γ responses between days 28 and 42 within each group. Data are presented as mean ± SD. Statistical significance was determined by one- or two-way ANOVA (** *p* < 0.01, **** *p* < 0.0001, ## *p* < 0.01, ### *p* < 0.001). Asterisks (*) denote comparison to the PC group; hash symbols (#) denote comparison to the NC group.

ELISpot assay quantified IFN-γ-secreting splenocytes (SFCs per 10^6^ cells) from immunized mice. At both day 28 and day 42 post-immunization, the two recombinant adenovirus vaccine groups and the inactivated vaccine group all elicited significantly higher numbers of IFN-γ^+^ SFCs compared to the NC group (*p* < 0.01; Figure 3A,B). Notably, the response induced by rAdV-I E0+I E2 was significantly stronger than that of the PC group at day 28 (*p* < 0.01). By day 42, both recombinant adenovirus groups maintained significantly elevated responses compared to the NC and PC groups (*p* < 0.01; Figure 3B). A longitudinal analysis further revealed a significant increase in IFN-γ production between day 28 and 42 within the rAdV-I E0+I E2 and rAdV-I E2+II E2 groups (*p* < 0.0001; Figure 3C), indicating a sustained and enhancing cellular immune response. Collectively, these data demonstrate that the adenoviral-vectored vaccines, particularly rAdV-I E0+I E2, robustly and durably stimulate antigen-specific IFN-γ production.

### 3.3. Adenoviral-Vectored Vaccines Induce Potent Humoral Immunity with Divergent Polarization

**Figure 4:**
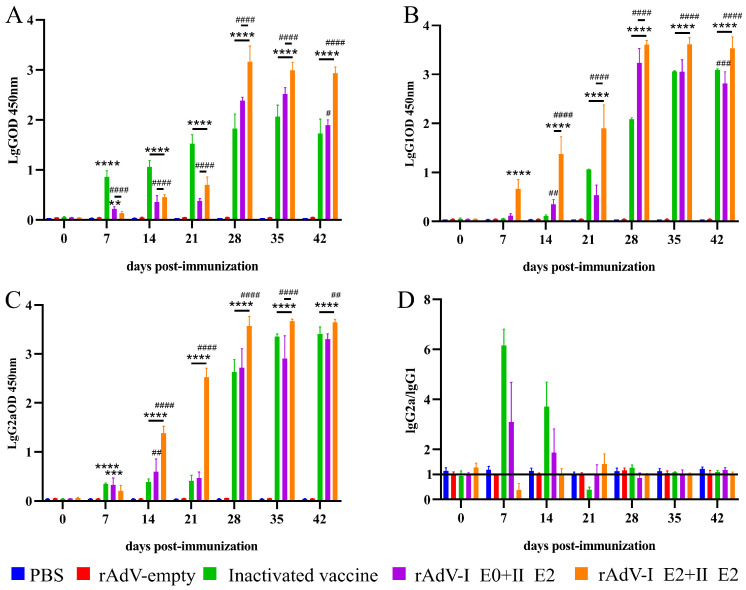
Recombinant adenovirus vaccines elicit robust and sustained BVDV-specific antibody responses. BALB/c mice were immunized with either rAdV-I E0+I E2 or rAdV-I E2+II E2, with a commercial inactivated vaccine as the PC and rAdV-empty as the NC. Serum was collected at indicated time points post-immunization. BVDV-specific antibodies were quantified by ELISA. (**A**–**C**) Kinetics of BVDV-specific total IgG (**A**), IgG1 (**B**), and IgG2a (**C**) antibodies. (**D**) Temporal change in the IgG2a/IgG1 ratio, indicating the Th1/Th2 bias. Data are presented as mean ± SD. Statistical significance was determined by two-way ANOVA (** *p* < 0.01, *** *p* < 0.001, **** *p* < 0.0001; # *p* < 0.05, ## *p* < 0.01, ### *p* < 0.001, #### *p* < 0.0001). Asterisks (*) denote comparison to the PC group; hash symbols (#) denote comparison to the NC group.

To evaluate the humoral immunogenicity of the adenoviral-vectored vaccines (rAdV-I E0+I E2 and rAdV-I E2+II E2), we immunized BALB/c mice and measured BVDV-specific antibody responses by ELISA. A commercial inactivated vaccine served as the PC, and an rAdV-empty served as the NC. As shown in Figure 4A–C, both the inactivated vaccine and the adenoviral-vectored vaccines induced robust and sustained production of BVDV-specific IgG, IgG1, and IgG2a antibodies.

We further assessed the Th1/Th2 bias by calculating the IgG2a/IgG1 ratio (Figure 4D). The commercial inactivated vaccine predominantly induced a Th1-biased response (ratio > 1) at most time points. Notably, the two adenoviral vectors elicited distinct immune polarization patterns. The rAdV-I E0+I E2 vaccine induced a mixed response, with Th1 bias at days 7, 14, and 42, but a Th2 bias at the intervening time points. In contrast, the rAdV-I E2+II E2 vaccine consistently promoted a Th2-dominant response (ratio < 1), with only a brief, transient Th1 shift at day 21. These results demonstrate that the adenoviral-vectored vaccines are potent inducers of humoral immunity, but their capacity to polarize the T-helper response differs significantly.

### 3.4. Induction of Neutralizing Antibodies by Recombinant Adenovirus Vaccines in Mice

**Figure 5:**
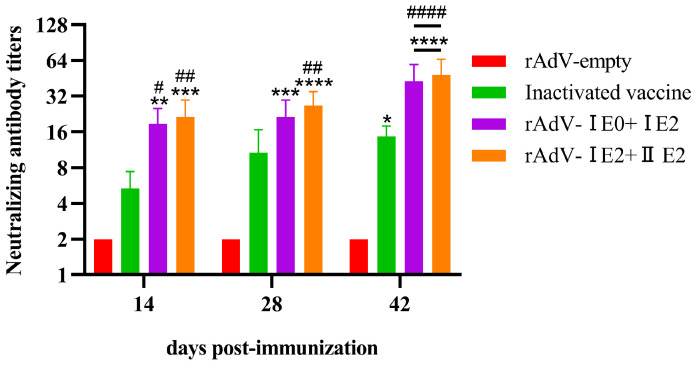
Recombinant adenovirus vaccines elicit potent and rapid neutralizing antibodies against BVDV. BALB/c mice were immunized with either rAdV-I E0+I E2 or rAdV-I E2+II E2 vaccine. A commercial inactivated vaccine and rAdV-empty served as PC and NC, respectively. Serum samples were collected at days 14, 28, and 42 post-immunization. BVDV-specific neutralizing antibody titers were determined by VNT. Data are presented as mean ± SD. Statistical significance was determined by two-way ANOVA (* *p* < 0.05, ** *p* < 0.01, *** *p* < 0.001, **** *p* < 0.0001; # *p* < 0.05, ## *p* < 0.01, #### *p* < 0.0001). Asterisks (*) denote comparison to the PC group; hash symbols (#) denote comparison to the NC group.

The neutralizing antibody response to recombinant adenovirus vaccines was evaluated in BALB/c mice, using rAdV-empty and a commercial inactivated vaccine as NC and PC, respectively. Sera collected on days 14, 28, and 42 post-immunization were analyzed for BVDV-specific antibodies by VNT. As shown in Figure 5, both the rAdV-I E0+I E2 and rAdV-I E2+II E2 vaccines induced significantly higher neutralizing antibody levels than the NC at all timepoints (*p* < 0.01). Compared to the PC, both recombinant vaccines elicited significantly higher neutralizing titers on days 14 and 42 (*p* < 0.05), while only the rAdV-I E2+II E2 vaccine maintained this superior response at day 28 (*p* < 0.01). These findings demonstrate that the recombinant adenovirus vaccines, particularly rAdV-I E2+II E2, provoke a faster and more potent neutralizing antibody response than the commercial inactivated vaccine.

### 3.5. Protection Against BVDV Infection by Recombinant Adenovirus Vaccines in Mice

**Figure 6:**
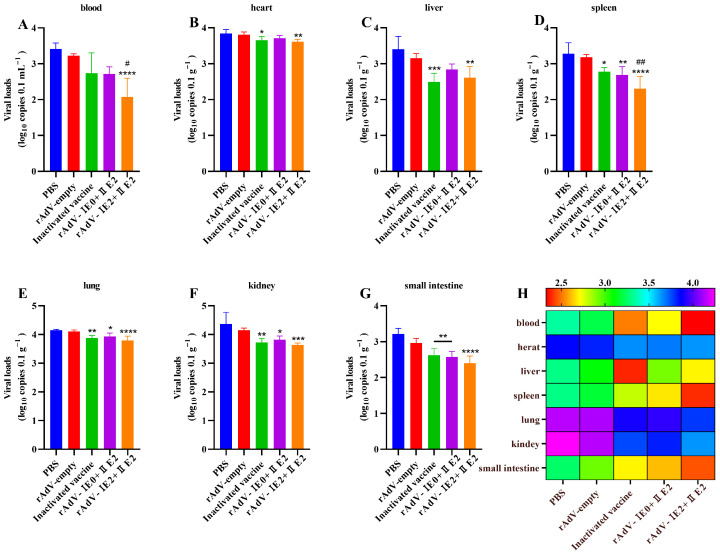
Recombinant adenovirus vaccines significantly reduce tissue viral loads following BVDV challenge. BALB/c mice were immunized with either rAdV-I E0+I E2 or rAdV-I E2+II E2 vaccine. A commercial inactivated vaccine and rAdV-empty served as PC and NC, respectively. Mice were challenged with BVDV on day 42 post-primary immunization. On day 14 post-challenge, tissues including blood (**A**), heart (**B**), liver (**C**), spleen (**D**), lungs (**E**), kidneys (**F**), and small intestine (**G**) were harvested. Viral RNA was extracted, and BVDV viral loads were quantified by RT-qPCR. (**H**) Heat map of viral load for each organization. Data are presented as mean ± SD. Statistical significance was determined by one-way ANOVA (* *p* < 0.05, ** *p* < 0.01, *** *p* < 0.001, **** *p* < 0.0001; # *p* < 0.05, ## *p* < 0.01). Asterisks (*) denote comparison to the PC group; hash symbols (#) denote comparison to the NC group.

To evaluate vaccine protection, viral loads in blood and multiple tissues (heart, liver, spleen, lungs, kidneys, and small intestine) were quantified by RT-qPCR at 14 days post-BVDV infection. Compared to the negative control (NC), all vaccinated groups significantly suppressed viral replication. Mice immunized with rAdV-I E0+I E2 exhibited reduced viral loads in the spleen, lungs, kidneys, and small intestine (Figure 6D–G; *p* < 0.05). The rAdV-I E2+II E2 vaccine conferred superior efficacy, significantly lowering viral loads across all tissues examined (Figure 6A–G; *p* < 0.01). Notably, viral loads in the blood and spleen of this group were also lower than those in the positive control (PC) group (Figure 6A,D; *p* < 0.05). Furthermore, the commercial inactivated vaccine provided substantial protection, with significantly decreased viral loads in the heart, liver, lungs, kidneys, and small intestine relative to the NC group (Figure 6B–G; *p* < 0.05). Collectively, these results demonstrate that while all vaccines were effective, the rAdV-I E2+II E2 vaccine induced the most robust and comprehensive protection against BVDV replication.

### 3.6. Vaccination Protects Duodenal and Splenic Architecture from BVDV-Induced Damage

**Figure 7:**
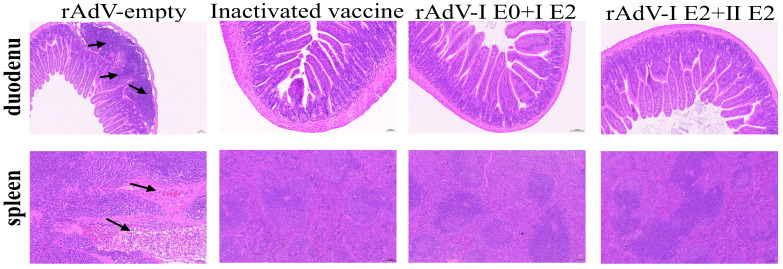
Adenovirus-vectored vaccination alleviates histopathological damage in BVDV-infected mice. BALB/c mice were challenged with BVDV on day 42 post-immunization. Tissues (duodenum and spleen) were harvested on day 14 post-challenge and analyzed by H&E staining (scale bar, 100 µm).

To evaluate the protective immunity conferred by the recombinant adenovirus vaccines against BVDV challenge, a histopathological examination was performed on day 14 post-challenge. The results showed that all vaccinated groups maintained well-preserved architecture in both the duodenum and spleen (Figure 7). In contrast, the NC group exhibited marked pathological damage, characterized by inflammatory cell infiltration in the lamina propria and mild submucosal congestion in the duodenum, as well as noticeable congestion in the spleen (indicated by black arrows).

## 4. Discussion

The development of novel and effective vaccines is paramount for controlling BVDV. While previous studies have utilized adenoviral vectors to express single BVDV antigens like E2 [21] or C [22], and protein-based vaccines focusing on E2 or E0 [15,16,17], our study advances the field by constructing and characterizing two novel bicistronic vectors co-expressing E0 and E2 from BVDV-1 or E2 proteins from both BVDV-1 and BVDV-2 genotypes. This design aims to harness potential synergistic effects for broader and more potent immunity. The successful packaging and confirmed genetic stability of our vaccines across serial passages are consistent with the known robustness of the adenovirus platform, but in this study, we demonstrate this stability for more complex bicistronic constructs, ensuring consistent immunogen delivery.

The potent Th1-skewed cellular immune response elicited by our vaccines, particularly rAdV-I E0+I E2, aligns with the established capacity of adenoviral vectors to stimulate T-cell immunity. However, the sustained and significantly increasing IFN-γ production we observed between day 28 and 42 goes beyond the responses typically reported for inactivated vaccines or even some single-antigen adenovirus constructs [21,22]. This underscores the potential of our bicistronic design to drive a more durable T-cell response, which is crucial for long-term protection against viral infections.

The most direct evidence of our vaccine’s enhanced immunogenicity in this model is reflected in the humoral response. The rAdV-I E2+II E2 vaccine induced neutralizing antibody (NAb) titers that were significantly higher and developed more rapidly than those elicited by the commercial inactivated vaccine. Moreover, the NAb levels achieved appeared comparable or superior to those reported for other BVDV subunit platforms, such as E2-based or virus-like particle (VLP) vaccines [17,33,34], which is consistent with the recognized capacity of adenoviral vectors to elicit humoral immunity. Notably, our bicistronic vaccines further modulated the cellular immune landscape, inducing distinct patterns of T-helper cell polarization. Specifically, we observed a mixed response from rAdV-I E0+I E2 versus a Th2-bias from rAdV-I E2+II E2. This suggests that the specific antigen combination within a bicistronic vector may actively influence immune polarization, a finding that merits further investigation to elucidate the underlying mechanisms.

The comprehensive protection conferred by rAdV-I E2+II E2, resulting in reduced viral loads across all tissues, demonstrates a clear advantage. Previous challenge studies using VLPs or inactivated vaccines have often shown partial protection, primarily reducing clinical signs but not always achieving significant viral clearance in all organs [24,34]. In contrast, our rAdV-I E2+II E2 vaccine’s ability to significantly lower viral loads in the blood and spleen below the level of the inactivated vaccine group sets a new benchmark in this murine model. Furthermore, the excellent correlation between strong Nab titers, reduced viral replication, and the preservation of tissue architecture provides a more complete and mechanistically supported chain of evidence for efficacy than viral load reduction alone.

In this study, we developed and evaluated two novel bicistronic adenoviral vectors co-expressing BVDV antigens. Our data demonstrate that immunization with these constructs, particularly rAdV-I E2+II E2, elicits a stronger and more durable immune response than a conventional inactivated vaccine, as evidenced by higher neutralizing antibody titers, a sustained Th1-skewed cellular response, and distinct immune polarization patterns. Critically, this enhanced immunogenicity translates into superior protection in vivo, with significantly reduced viral loads and preserved tissue architecture following BVDV challenge. It is important to note that these promising results were obtained in a murine model. While they provide strong proof-of-concept, further evaluation in bovine hosts is essential and warranted to confirm their protective efficacy, safety, and broader applicability in the natural target species.

## Figures and Tables

**Table 1:** Primers used in this study.

Primer Name	Primer Sequences (5′–3′)	Product Size (bp)
I E0+I E2-F	ATGGAGAACATCACACAGTGGAACC	1755
I E0+I E2-R	CGCTAATGACCATGTAGGTGTAGTC	
I E2+II E2-F	CTGAGTGTAAGGAGGGCTTT	2301
I E2+II E2-R	GGGCCTTCTGCTCGCTAATGA	

**Table 2:** Immunization strategies.

Groups (*n* = 10)	Dose (PFU)	Immunization Time (d)
rAdV-I E0+I E2	10^7^	0, 21
rAdV-I E2+II E2	10^7^	0, 21
rAdV-empty (negative control, NC)	10^7^	0, 21
Inactivated vaccine (positive control, PC)	100 μL	0, 21
PBS	100 μL	0, 21

## Data Availability

The original contributions presented in this study are included in the article/Supplementary Materials. Further inquiries can be directed to the corresponding authors.

## Supplementary Materials

The following supporting information can be downloaded at https://www.mdpi.com/article/10.3390/microorganisms14020378/s1, Figure S1. Restriction Analysis of Recombinant Adenoviral Shuttle and Viral Plasmids. (A) Verification of the shuttle plasmids pAdTrack-I E0+I E2 and pAdTrack-I E2+II E2 through double digestion with SalI/EcoRV and SalI/XhoI, respectively. (B) Linearization of the recombinant adenoviral plasmids pAdEasy-I E0+I E2 and pAdEasy-I E2+II E2 by digestion with the unique restriction enzyme PacI.

## Abbreviations

The following abbreviations are used in this manuscript:

BVDV	Bovine viral diarrhea virus
PI	Persistently infected
CPE	Cytopathic effects
ELISA	Enzyme-Linked Immunosorbent Assay
ELISpot	Enzyme-linked Immunospot
ConA	Concanavalin A
PBS	Phosphate-buffered saline
SFCs	Spot-forming cells
IFN-γ	Interferon-γ
SDs	Standard deviations
