# Supplementary material for: A Novel Bicistronic Adenovirus Vaccine Elicits Superior and Comprehensive Protection Against BVDV
Source: Microorganisms. 2026 Feb 5;14(2):378. doi: 10.3390/microorganisms14020378 (PMC12942745; doi:10.3390/microorganisms14020378)
Supplement: Supplementary file 1 [file microorganisms-14-00378-s001.zip › microorganisms-4098028-supplementary.pdf]

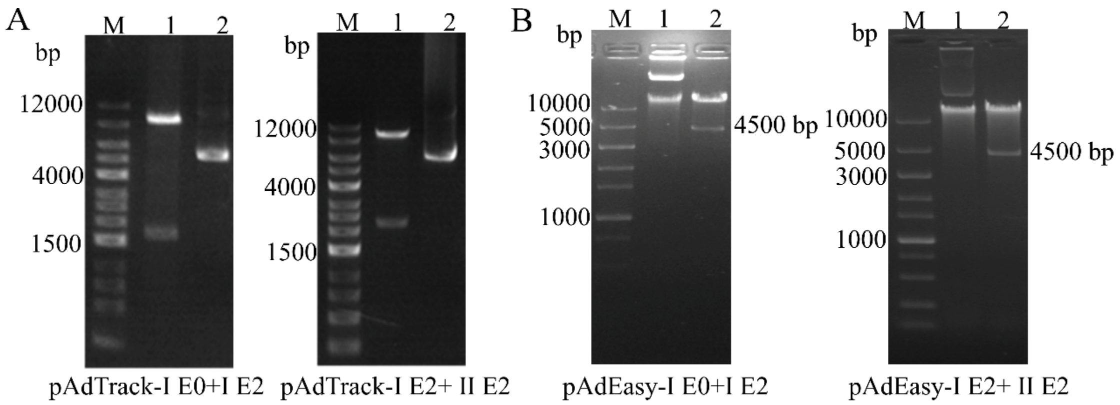

Figure S1. Restriction Analysis of Recombinant Adenoviral Shuttle and Viral Plasmids. (A) Verification of the shuttle plasmids pAdTrack-I E0+I E2 and pAdTrack-I E2+II E2 through double digestion with SalI/EcoRV and SalI/XhoI, respectively. (B) Linearization of the recombinant adenoviral plasmids pAdEasy-I E0+I E2 and pAdEasy-I E2+II E2 by digestion with the unique restriction enzyme PacI.
